# Supplementary material for: MicroRNA-27b-3p Targets the Myostatin Gene to Regulate Myoblast Proliferation and Is Involved in Myoblast Differentiation
Source: Cells. 2021 Feb 17;10(2):423. doi: 10.3390/cells10020423 (PMC7922189; doi:10.3390/cells10020423)
Supplement: Supplementary file 1 [file cells-10-00423-s001.zip › cells-1048437-Supplementary Materials/S1/miR-27b-3p inhibitor and inhibitor NC/miR-27b-3p inhibitor NC-1.pdf]

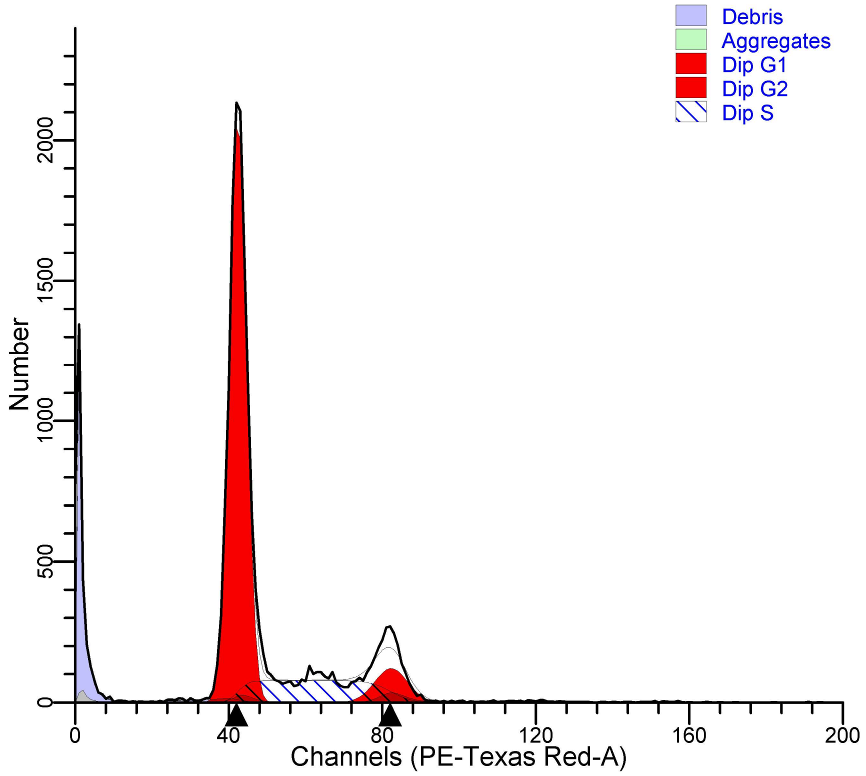

File analyzed: s7_in1_004.fcs
Date analyzed: 19-Jan-2021
Model: 1DA0n_DSD
Analysis type: Manual analysis

Ploidy Mode: First cycle is diploid

Diploid: 100.00 %
Dip G1: 72.66 % at 42.40
Dip G2: 8.16 % at 82.27
Dip S: 19.17 %   G2/G1: 1.94
%CV: 5.21

Total S-Phase: 19.17 %
Total B.A.D.: 3.26 %

Debris: 13.17 %
Aggregates: 4.19 %
Modeled events: 19340
All cycle events: 15983
Cycle events per channel: 391
RCS: 2.992

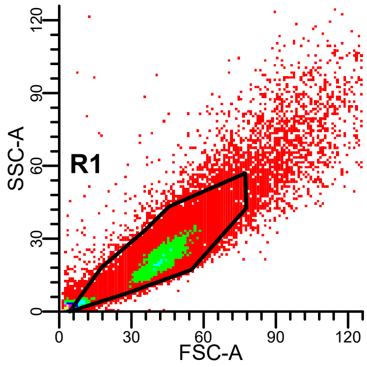

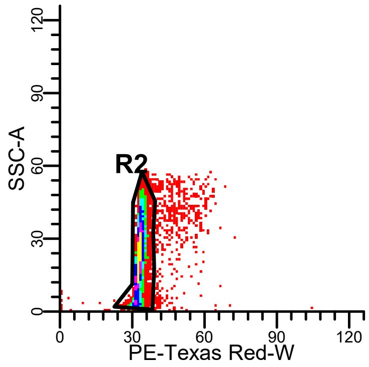

ModFitLT V3.1(Win32)
