# Supplementary material for: MicroRNA-27b-3p Targets the Myostatin Gene to Regulate Myoblast Proliferation and Is Involved in Myoblast Differentiation
Source: Cells. 2021 Feb 17;10(2):423. doi: 10.3390/cells10020423 (PMC7922189; doi:10.3390/cells10020423)
Supplement: Supplementary file 1 [file cells-10-00423-s001.zip › cells-1048437-Supplementary Materials/S1/miR-27b-3p inhibitor and inhibitor NC/miR-27b-3p inhibitor NC-2.pdf]

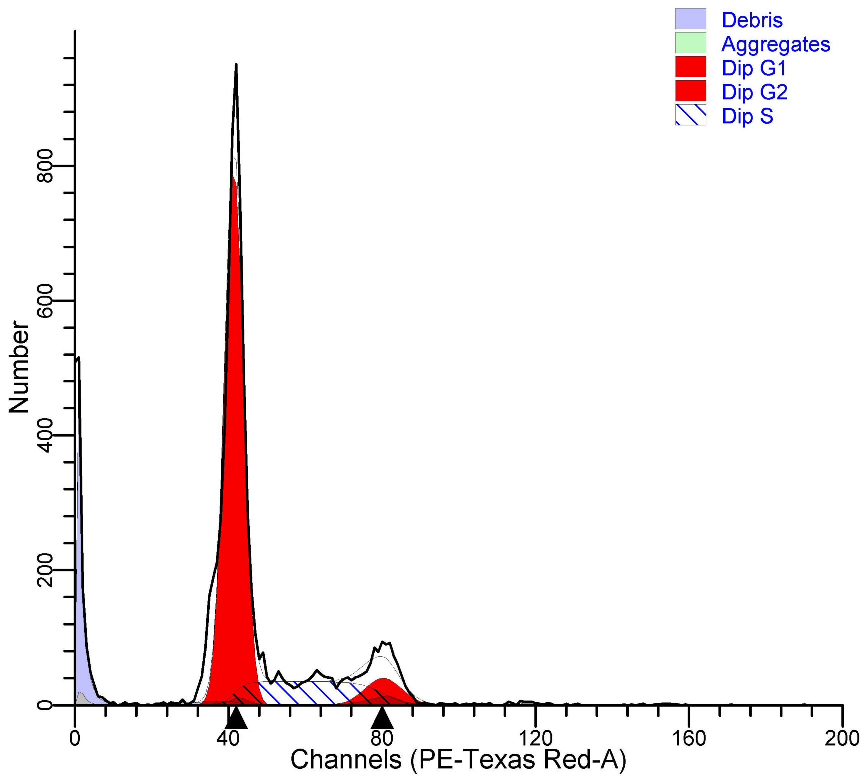


File analyzed: s7_in2_005.fcs
Date analyzed: 19-Jan-2021
Model: 1DA0n_DSD
Analysis type: Manual analysis

Ploidy Mode: First cycle is diploid

Diploid: 100.00 %
Dip G1: 73.17 % at 41.40
Dip G2: 7.12 % at 80.32
Dip S: 19.70 % G2/G1: 1.94
%CV: 6.01

Total S-Phase: 19.70 %
Total B.A.D.: 3.84 %

Debris: 12.13 %
Aggregates: 4.54 %
Modeled events: 8206
All cycle events: 6837
Cycle events per channel: 171
RCS: 3.322

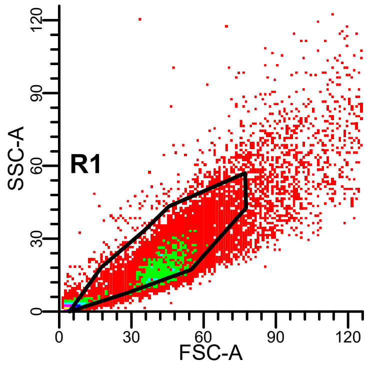


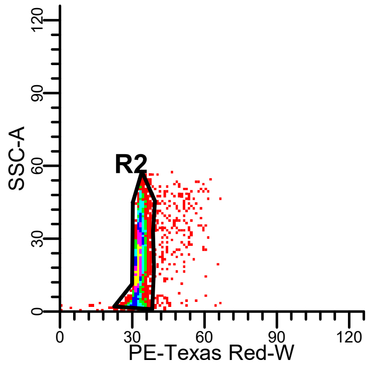


ModFitLT V3.1(Win32)
