# Supplementary material for: MicroRNA-27b-3p Targets the Myostatin Gene to Regulate Myoblast Proliferation and Is Involved in Myoblast Differentiation
Source: Cells. 2021 Feb 17;10(2):423. doi: 10.3390/cells10020423 (PMC7922189; doi:10.3390/cells10020423)
Supplement: Supplementary file 1 [file cells-10-00423-s001.zip › cells-1048437-Supplementary Materials/S1/miR-27b-3p inhibitor and inhibitor NC/miR-27b-3p inhibitor NC-3.pdf]

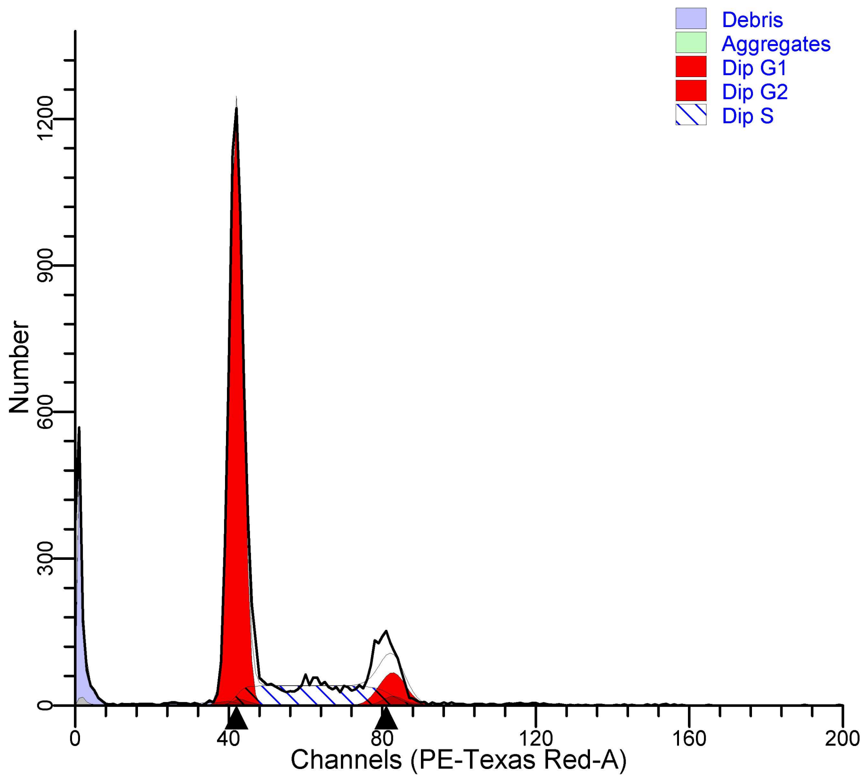

File analyzed: s7_in3_006.fcs
Date analyzed: 19-Jan-2021
Model: 1DA0n_DSD
Analysis type: Manual analysis

Ploidy Mode: First cycle is diploid

Diploid: 100.00 %
Dip G1: 71.17 % at 41.94
Dip G2: 7.68 % at 82.63
Dip S: 21.16 %   G2/G1: 1.97
%CV: 4.20

Total S-Phase: 21.16 %
Total B.A.D.: 2.96 %

Debris: 11.86 %
Aggregates: 4.04 %
Modeled events: 9044
All cycle events: 7606
Cycle events per channel: 182
RCS: 2.553

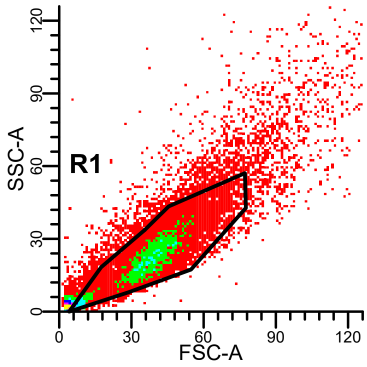

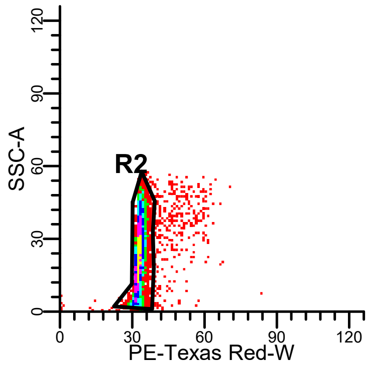

ModFitLT V3.1(Win32)
