# Supplementary material for: MicroRNA-27b-3p Targets the Myostatin Gene to Regulate Myoblast Proliferation and Is Involved in Myoblast Differentiation
Source: Cells. 2021 Feb 17;10(2):423. doi: 10.3390/cells10020423 (PMC7922189; doi:10.3390/cells10020423)
Supplement: Supplementary file 1 [file cells-10-00423-s001.zip › cells-1048437-Supplementary Materials/S1/miR-27b-3p mimic and mimic NC/miR-27b-3p mimics NC-1.pdf]

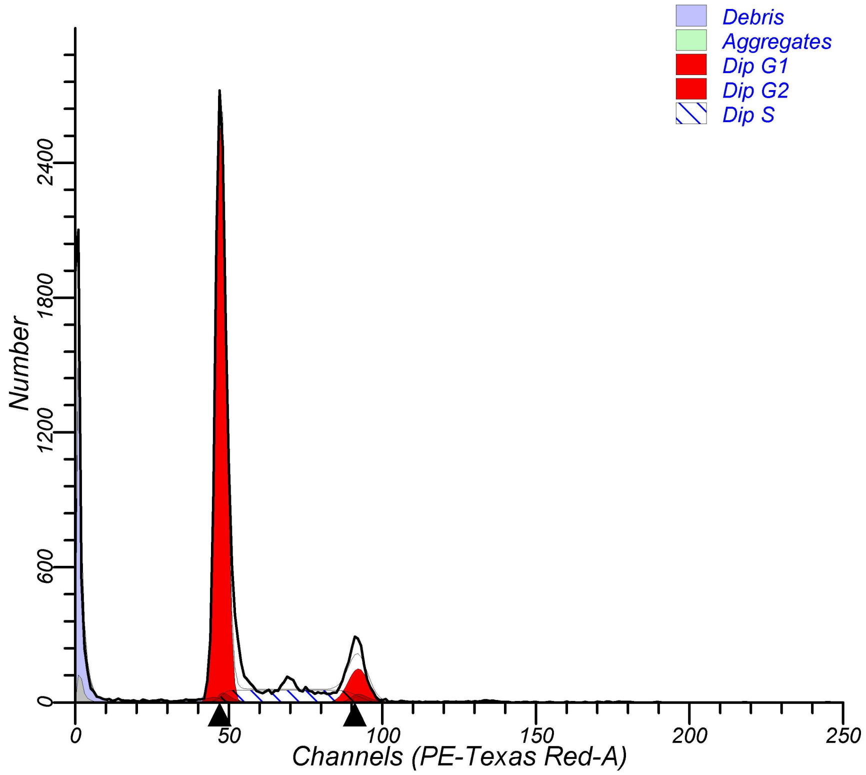

File analyzed: S5_mn1_004.fcs
Date analyzed: 28-Oct-2019
Model: 1DA0n_DSD
Analysis type: Manual analysis

Ploidy Mode: First cycle is diploid

Diploid: 100.00 %
Dip G1: 75.56 % at 47.48
Dip G2: 8.21 % at 92.10
Dip S: 16.24 % G2/G1: 1.94
%CV: 3.57

Total S-Phase: 16.24 %
Total B.A.D.: 3.89 %

Debris: 17.92 %
Aggregates: 5.14 %
Modeled events: 19709
All cycle events: 15163
Cycle events per channel: 332
RCS: 5.614

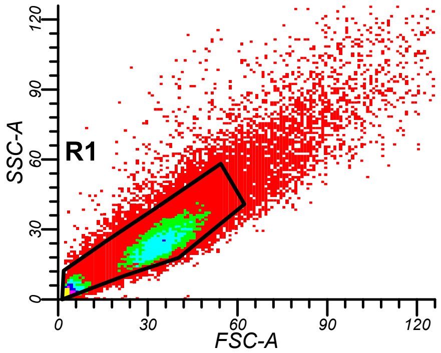

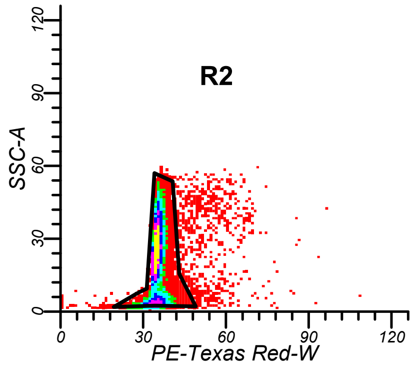

ModFitLT V3.1(Win32)
