# Supplementary material for: MicroRNA-27b-3p Targets the Myostatin Gene to Regulate Myoblast Proliferation and Is Involved in Myoblast Differentiation
Source: Cells. 2021 Feb 17;10(2):423. doi: 10.3390/cells10020423 (PMC7922189; doi:10.3390/cells10020423)
Supplement: Supplementary file 1 [file cells-10-00423-s001.zip › cells-1048437-Supplementary Materials/S1/miR-27b-3p mimic and mimic NC/miR-27b-3p mimics NC-2.pdf]

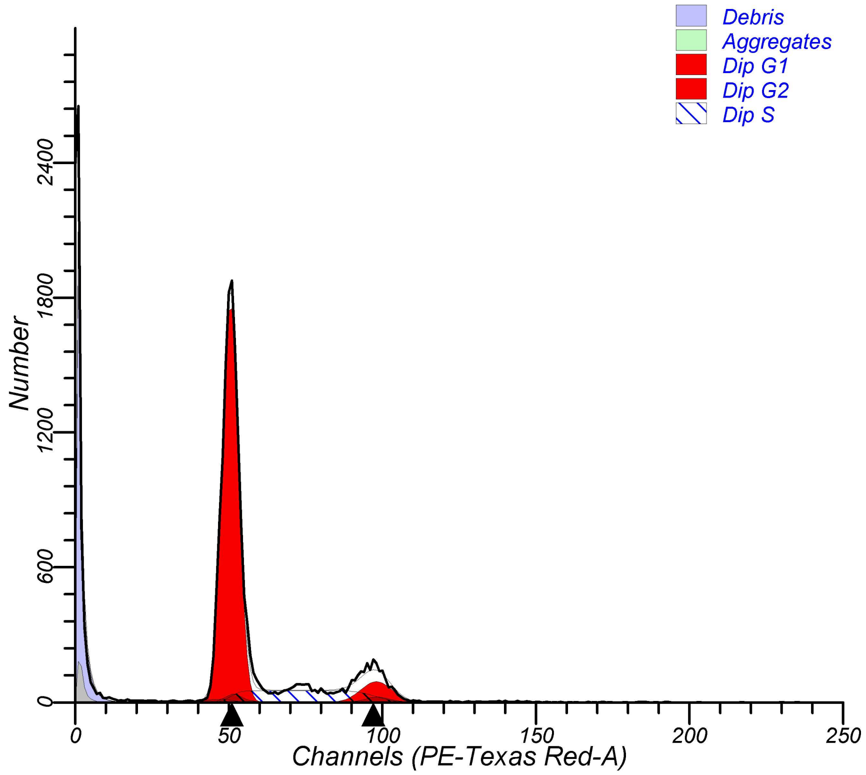

File analyzed: S5_MN2_005.fcs
Date analyzed: 28-Oct-2019
Model: 1DA0n_DSD
Analysis type: Manual analysis

Ploidy Mode: First cycle is diploid

Diploid: 100.00 %
Dip G1: 76.14 % at 50.53
Dip G2: 7.68 % at 98.03
Dip S: 16.18 %   G2/G1: 1.94
%CV: 5.17

Total S-Phase: 16.18 %
Total B.A.D.: 4.06 %

Debris: 20.54 %
Aggregates: 5.63 %
Modeled events: 20858
All cycle events: 15399
Cycle events per channel: 318
RCS: 3.202

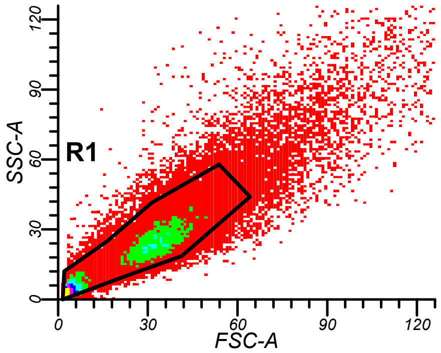

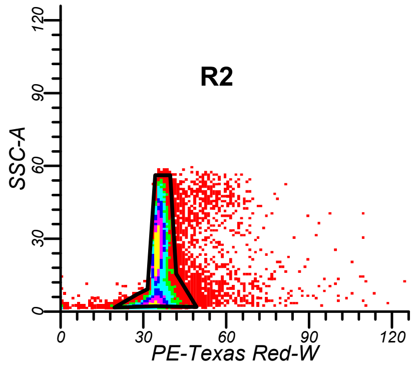

ModFitLT V3.1(Win32)
