# Supplementary material for: MicroRNA-27b-3p Targets the Myostatin Gene to Regulate Myoblast Proliferation and Is Involved in Myoblast Differentiation
Source: Cells. 2021 Feb 17;10(2):423. doi: 10.3390/cells10020423 (PMC7922189; doi:10.3390/cells10020423)

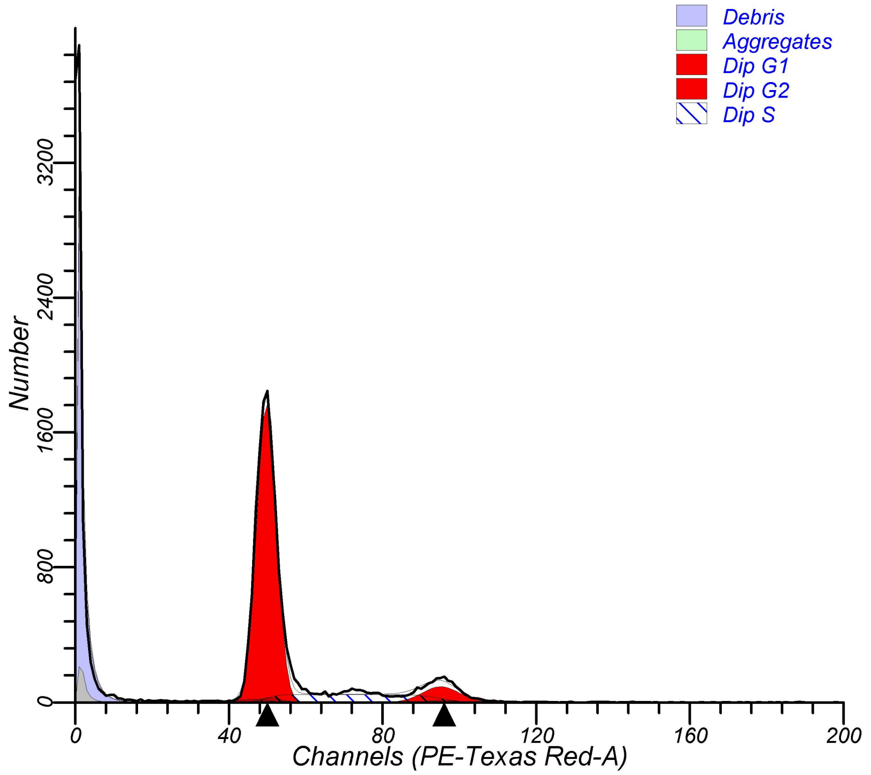

File analyzed: S5_MN3_006.fcs
Date analyzed: 28-Oct-2019
Model: 1DA0n_DSD
Analysis type: Manual analysis

Ploidy Mode: First cycle is diploid

Diploid: 100.00 %
Dip G1: 77.52 % at 49.80
Dip G2: 7.78 % at 95.12
Dip S: 14.70 % G2/G1: 1.91
%CV: 5.09

Total S-Phase: 14.70 %
Total B.A.D.: 3.37 %

Debris: 28.80 %
Aggregates: 4.36 %
Modeled events: 21849
All cycle events: 14604
Cycle events per channel: 315
RCS: 4.906

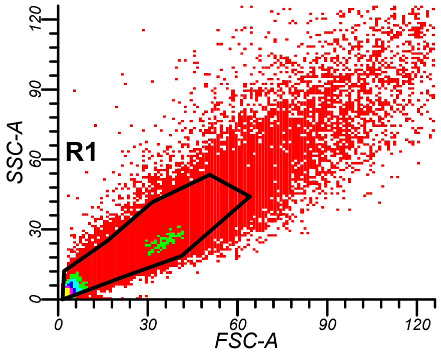

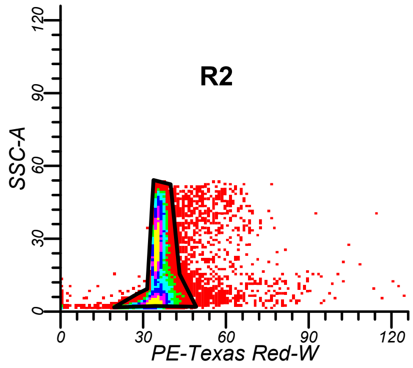

Supplement: Supplementary file 1 [file cells-10-00423-s001.zip › cells-1048437-Supplementary Materials/S1/miR-27b-3p mimic and mimic NC/miR-27b-3p mimics NC-3.pdf]
