# Supplementary material for: MicroRNA-27b-3p Targets the Myostatin Gene to Regulate Myoblast Proliferation and Is Involved in Myoblast Differentiation
Source: Cells. 2021 Feb 17;10(2):423. doi: 10.3390/cells10020423 (PMC7922189; doi:10.3390/cells10020423)

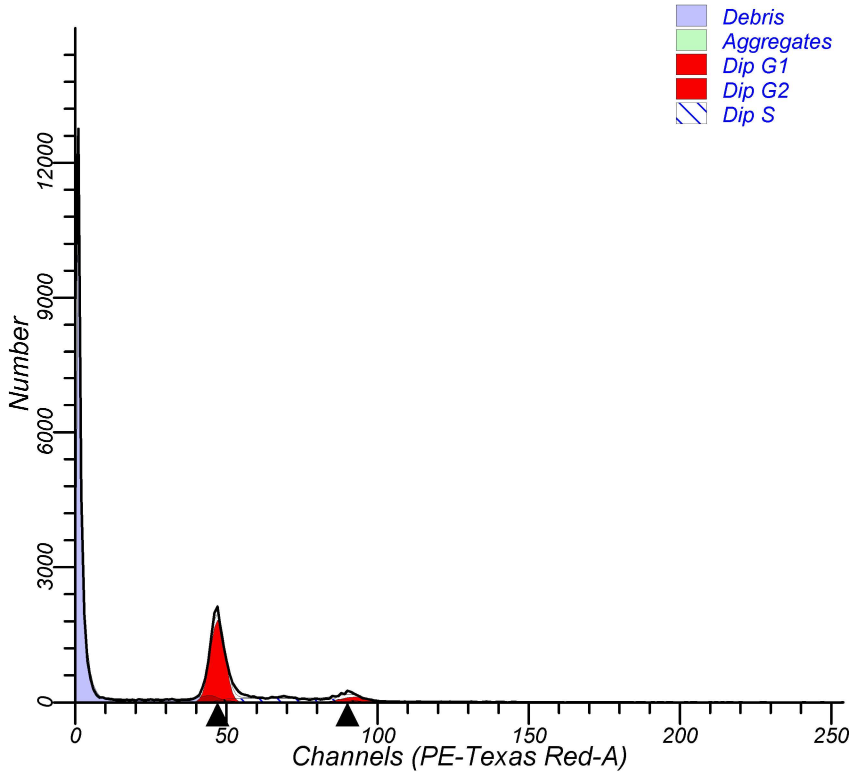

File analyzed: S5_M1_001.fcs
Date analyzed: 28-Oct-2019
Model: 1DA0n_DSD
Analysis type: Manual analysis

Ploidy Mode: First cycle is diploid

Diploid: 100.00 %
Dip G1: 67.79 % at 47.05
Dip G2: 8.62 % at 92.21
Dip S: 23.58 % G2/G1: 1.96
%CV: 5.05

Total S-Phase: 23.58 %
Total B.A.D.: 8.26 %

Debris: 61.64 %
Aggregates: 0.00 %
Modeled events: 42241
All cycle events: 16204
Cycle events per channel: 351
RCS: 6.253

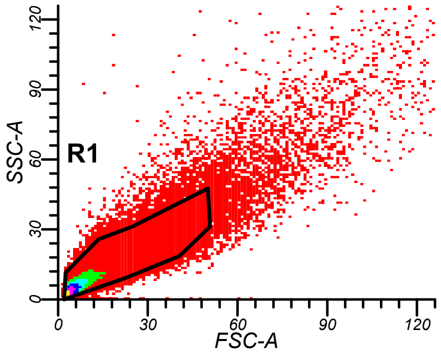

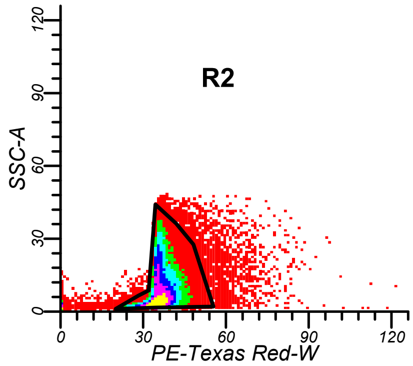

Supplement: Supplementary file 1 [file cells-10-00423-s001.zip › cells-1048437-Supplementary Materials/S1/miR-27b-3p mimic and mimic NC/miR-27b-3p mimics-1.pdf]
