# Supplementary material for: MicroRNA-27b-3p Targets the Myostatin Gene to Regulate Myoblast Proliferation and Is Involved in Myoblast Differentiation
Source: Cells. 2021 Feb 17;10(2):423. doi: 10.3390/cells10020423 (PMC7922189; doi:10.3390/cells10020423)
Supplement: Supplementary file 1 [file cells-10-00423-s001.zip › cells-1048437-Supplementary Materials/S1/miR-27b-3p mimic and mimic NC/miR-27b-3p mimics-2.pdf]

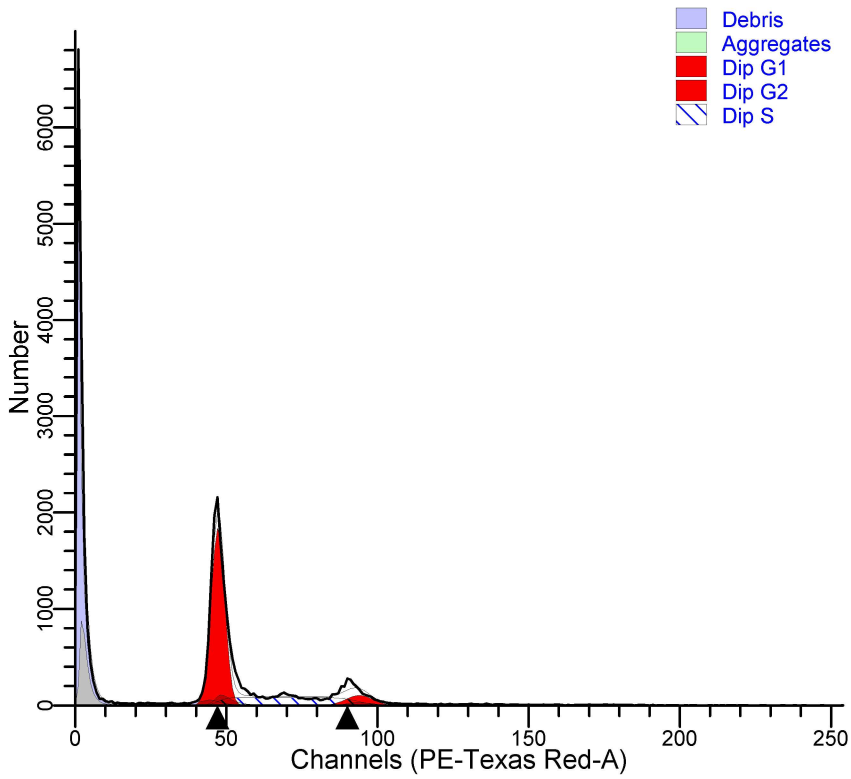

File analyzed: S5_M2_002.fcs
Date analyzed: 19-Jan-2021
Model: 1DA0n_DSD
Analysis type: Manual analysis

Ploidy Mode: First cycle is diploid

Diploid: 100.00 %
Dip G1: 67.60 % at 47.18
Dip G2: 7.54 % at 93.90
Dip S: 24.85 %   G2/G1: 1.99
%CV: 4.68

Total S-Phase: 24.85 %
Total B.A.D.: 10.56 %

Debris: 39.90 %
Aggregates: 13.24 %
Modeled events: 32508
All cycle events: 15233
Cycle events per channel: 319
RCS: 6.340

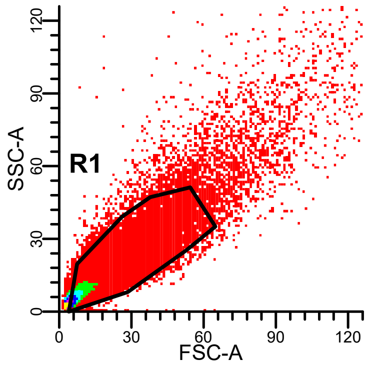

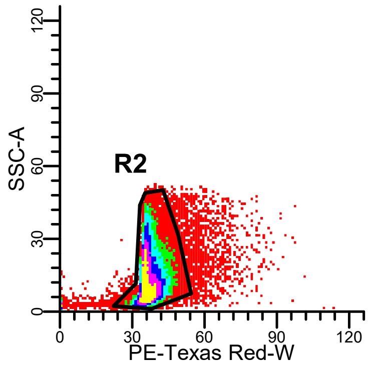

ModFitLT V3.1(Win32)
