# Supplementary material for: MicroRNA-27b-3p Targets the Myostatin Gene to Regulate Myoblast Proliferation and Is Involved in Myoblast Differentiation
Source: Cells. 2021 Feb 17;10(2):423. doi: 10.3390/cells10020423 (PMC7922189; doi:10.3390/cells10020423)
Supplement: Supplementary file 1 [file cells-10-00423-s001.zip › cells-1048437-Supplementary Materials/S2/pcDNA 3.1-MSTN and pcDNA 3.1/pcDNA 3.1-2.pdf]

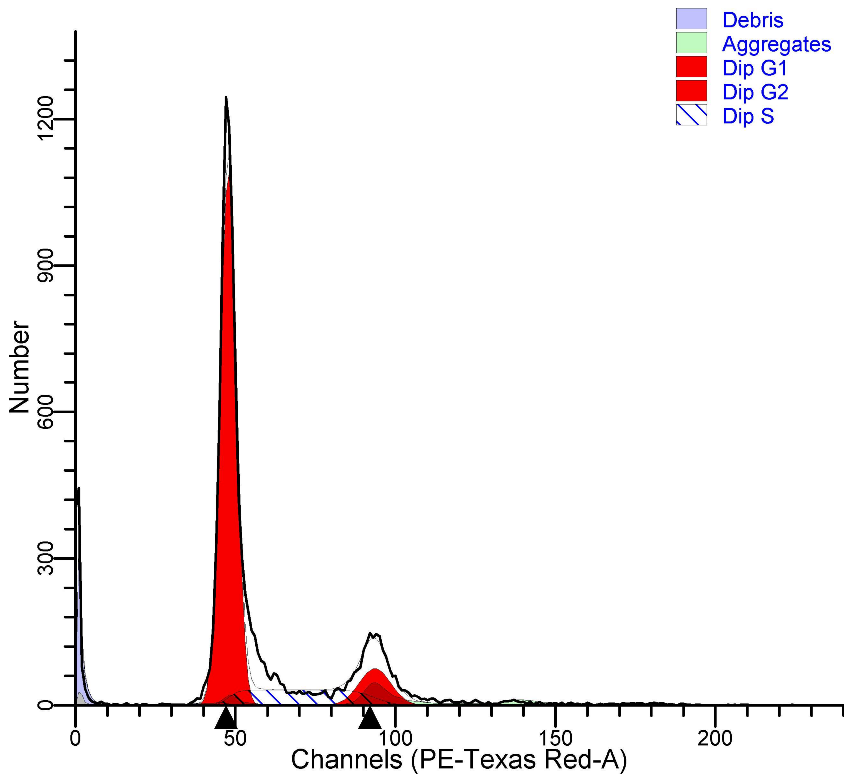

File analyzed: 005.fcs
Date analyzed: 9-Jul-2020
Model: 1DA0n_DSD
Analysis type: Manual analysis

Ploidy Mode: First cycle is diploid

Diploid: 100.00 %
Dip G1: 74.81 % at 47.75
Dip G2: 10.02 % at 93.59
Dip S: 15.17 %   G2/G1: 1.96
%CV: 5.27

Total S-Phase: 15.17 %
Total B.A.D.: 5.08 %

Debris: 6.20 %
Aggregates: 9.35 %
Modeled events: 11010
All cycle events: 9298
Cycle events per channel: 199
RCS: 4.444

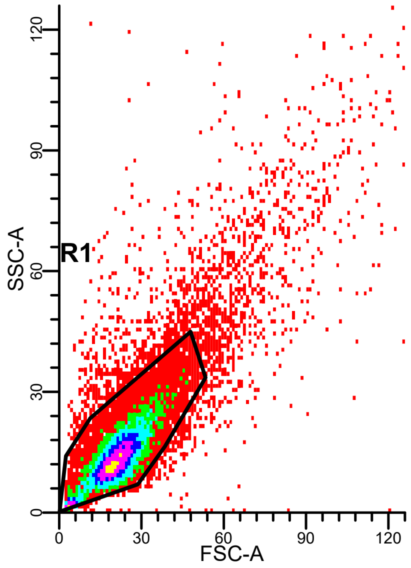

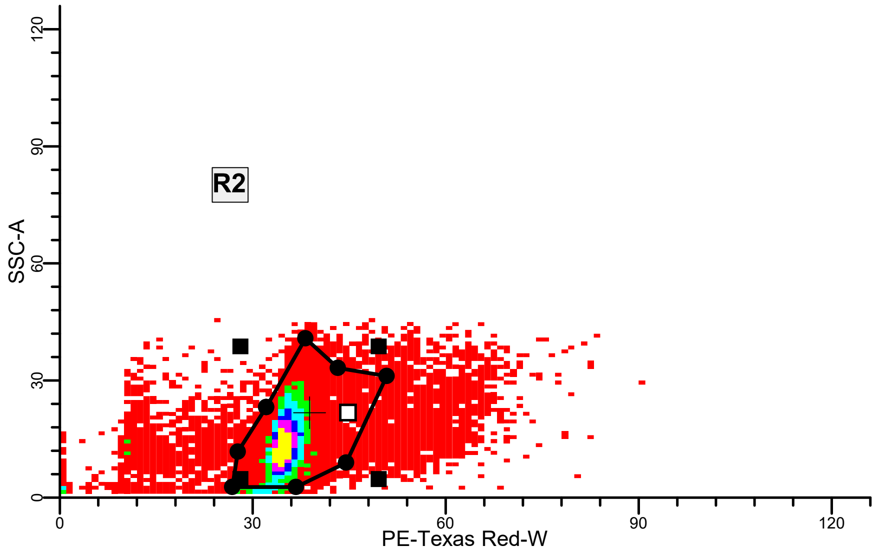

ModFitLT V3.1(Win32)
