# Supplementary material for: MicroRNA-27b-3p Targets the Myostatin Gene to Regulate Myoblast Proliferation and Is Involved in Myoblast Differentiation
Source: Cells. 2021 Feb 17;10(2):423. doi: 10.3390/cells10020423 (PMC7922189; doi:10.3390/cells10020423)
Supplement: Supplementary file 1 [file cells-10-00423-s001.zip › cells-1048437-Supplementary Materials/S2/pcDNA 3.1-MSTN and pcDNA 3.1/pcDNA 3.1-3.pdf]

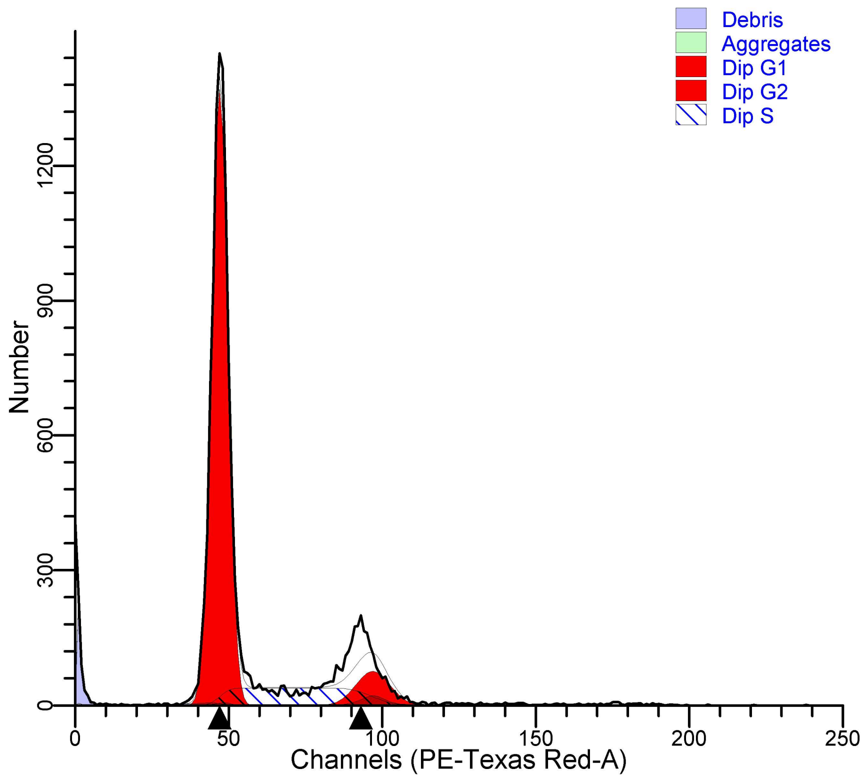

File analyzed: 006.fcs
Date analyzed: 19-Jan-2021
Model: 1DA0n_DSD
Analysis type: Manual analysis

Ploidy Mode: First cycle is diploid

Diploid: 100.00 %
Dip G1: 75.54 % at 47.03
Dip G2: 8.59 % at 96.88
Dip S: 15.87 %   G2/G1: 2.06
%CV: 5.69

Total S-Phase: 15.87 %
Total B.A.D.: 2.19 %

Debris: 4.29 %
Aggregates: 3.22 %
Modeled events: 13227
All cycle events: 12234
Cycle events per channel: 241
RCS: 3.463

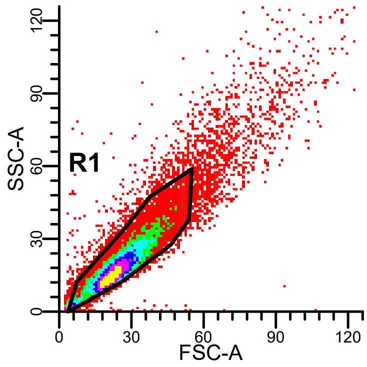

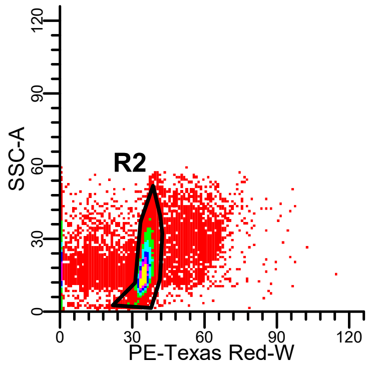

ModFitLT V3.1(Win32)
