# Supplementary material for: MicroRNA-27b-3p Targets the Myostatin Gene to Regulate Myoblast Proliferation and Is Involved in Myoblast Differentiation
Source: Cells. 2021 Feb 17;10(2):423. doi: 10.3390/cells10020423 (PMC7922189; doi:10.3390/cells10020423)
Supplement: Supplementary file 1 [file cells-10-00423-s001.zip › cells-1048437-Supplementary Materials/S2/pcDNA 3.1-MSTN and pcDNA 3.1/pcDNA 3.1-MSTN-1.pdf]

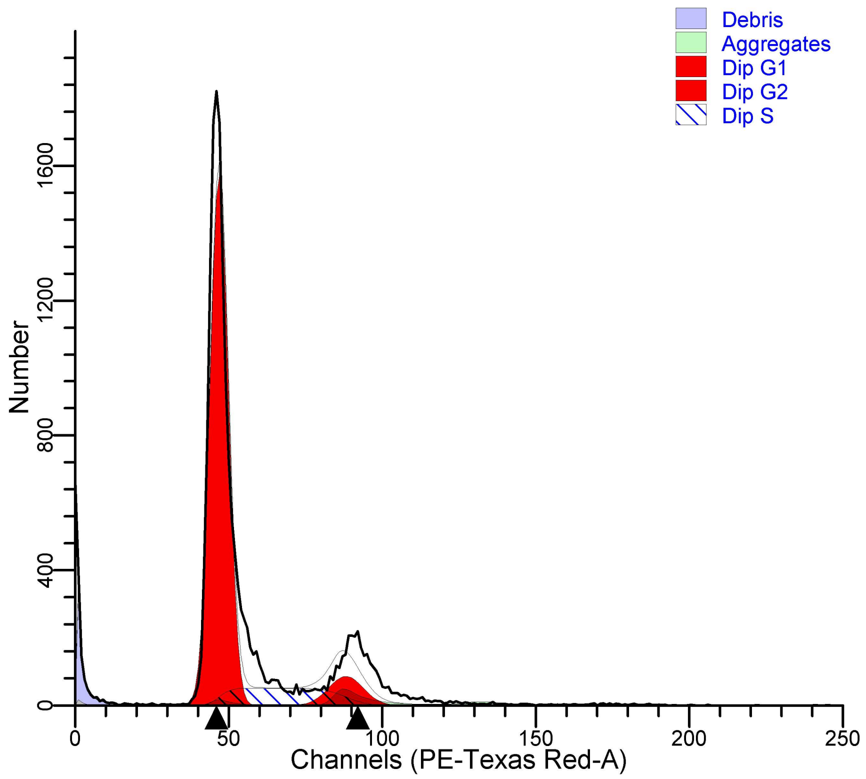

File analyzed: 001.fcs
Date analyzed: 9-Jul-2020
Model: 1DA0n_DSD
Analysis type: Manual analysis

Ploidy Mode: First cycle is diploid

Diploid: 100.00 %
Dip G1: 78.12 % at 46.88
Dip G2: 8.03 % at 88.13
Dip S: 13.85 % G2/G1: 1.88
%CV: 6.33

Total S-Phase: 13.85 %
Total B.A.D.: 3.58 %

Debris: 5.22 %
Aggregates: 6.57 %
Modeled events: 17063
All cycle events: 15050
Cycle events per channel: 356
RCS: 10.135

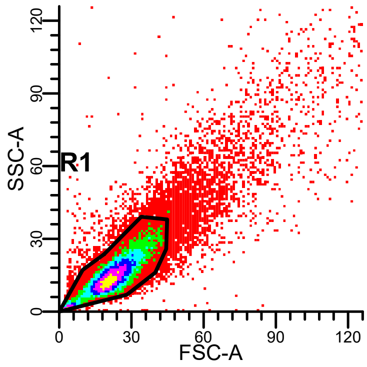

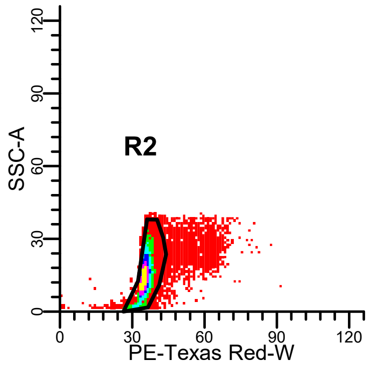

ModFitLT V3.1(Win32)
