# Supplementary material for: MicroRNA-27b-3p Targets the Myostatin Gene to Regulate Myoblast Proliferation and Is Involved in Myoblast Differentiation
Source: Cells. 2021 Feb 17;10(2):423. doi: 10.3390/cells10020423 (PMC7922189; doi:10.3390/cells10020423)
Supplement: Supplementary file 1 [file cells-10-00423-s001.zip › cells-1048437-Supplementary Materials/S2/pcDNA 3.1-MSTN and pcDNA 3.1/pcDNA 3.1-MSTN-3.pdf]

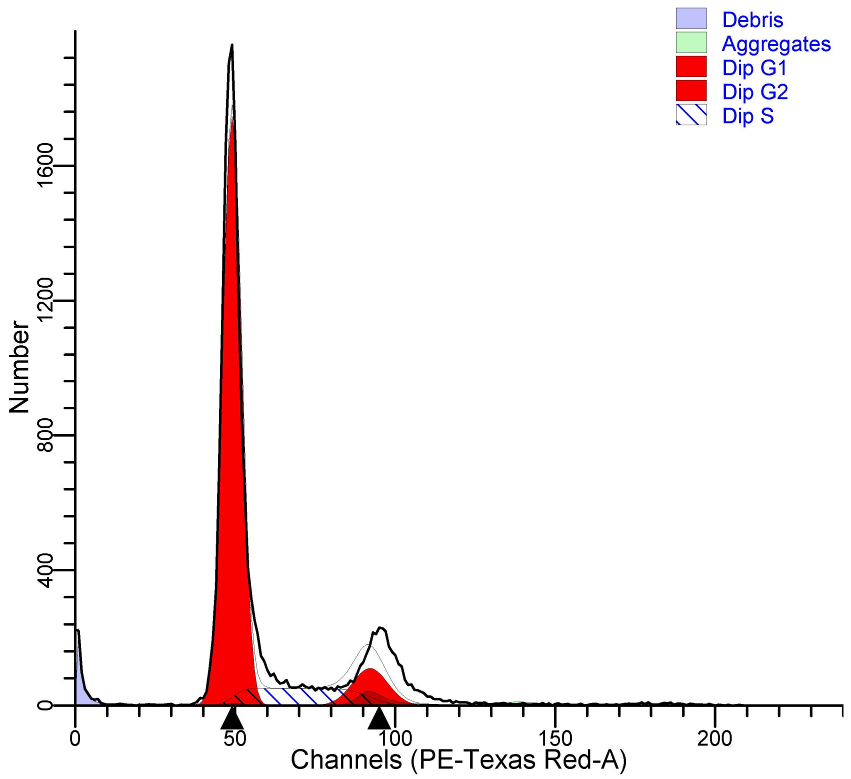

File analyzed: 003.fcs
Date analyzed: 9-Jul-2020
Model: 1DA0n_DSD
Analysis type: Manual analysis

Ploidy Mode: First cycle is diploid

Diploid: 100.00 %
Dip G1: 77.60 % at 49.00
Dip G2: 9.16 % at 92.12
Dip S: 13.24 %   G2/G1: 1.88
%CV: 5.97

Total S-Phase: 13.24 %
Total B.A.D.: 2.33 %

Debris: 3.08 %
Aggregates: 4.58 %
Modeled events: 17981
All cycle events: 16605
Cycle events per channel: 376
RCS: 7.756

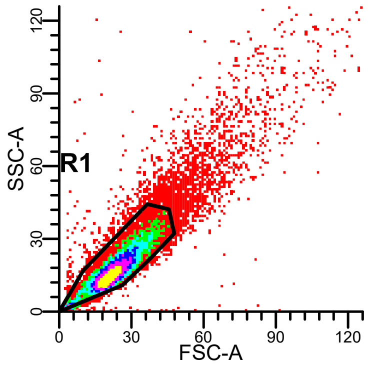

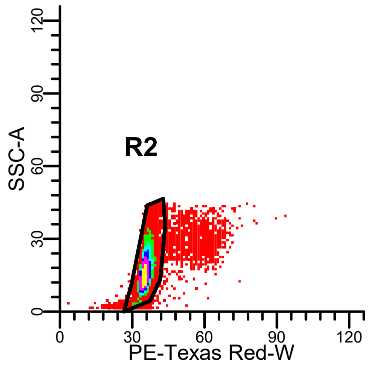

ModFitLT V3.1(Win32)
