# Supplementary material for: MicroRNA-27b-3p Targets the Myostatin Gene to Regulate Myoblast Proliferation and Is Involved in Myoblast Differentiation
Source: Cells. 2021 Feb 17;10(2):423. doi: 10.3390/cells10020423 (PMC7922189; doi:10.3390/cells10020423)

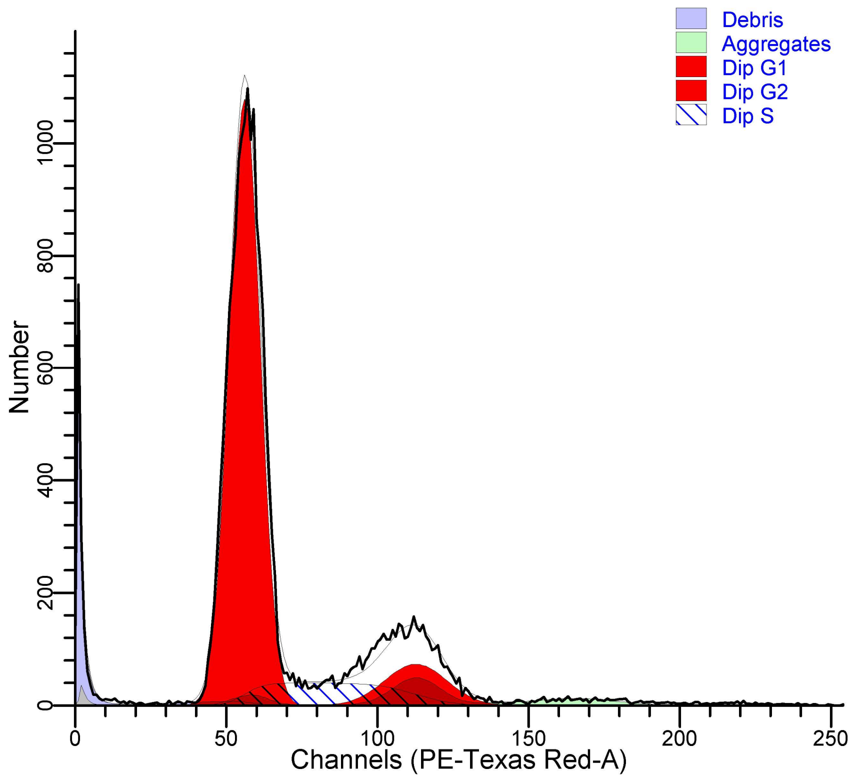

File analyzed: s7_GR1_004.fcs
Date analyzed: 19-Jan-2021
Model: 1DA0n_DSD
Analysis type: Manual analysis

Ploidy Mode: First cycle is diploid

Diploid: 100.00 %
Dip G1: 76.96 % at 56.03
Dip G2: 10.52 % at 112.62
Dip S: 12.52 %   G2/G1: 2.01
%CV: 9.10

Total S-Phase: 12.52 %
Total B.A.D.: 6.62 %

Debris: 7.60 %
Aggregates: 9.01 %
Modeled events: 21524
All cycle events: 17949
Cycle events per channel: 312
RCS: 2.523

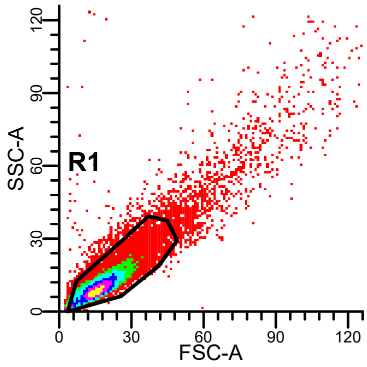

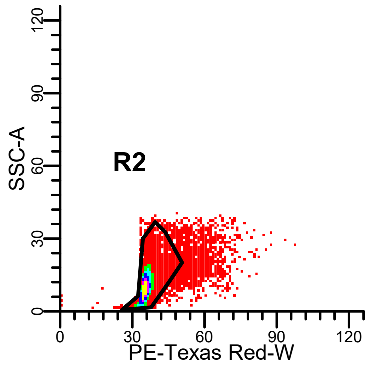

Supplement: Supplementary file 1 [file cells-10-00423-s001.zip › cells-1048437-Supplementary Materials/S2/siR-MSTN and siR-NC/siR-MSTN-1.pdf]
