# Supplementary material for: MicroRNA-27b-3p Targets the Myostatin Gene to Regulate Myoblast Proliferation and Is Involved in Myoblast Differentiation
Source: Cells. 2021 Feb 17;10(2):423. doi: 10.3390/cells10020423 (PMC7922189; doi:10.3390/cells10020423)

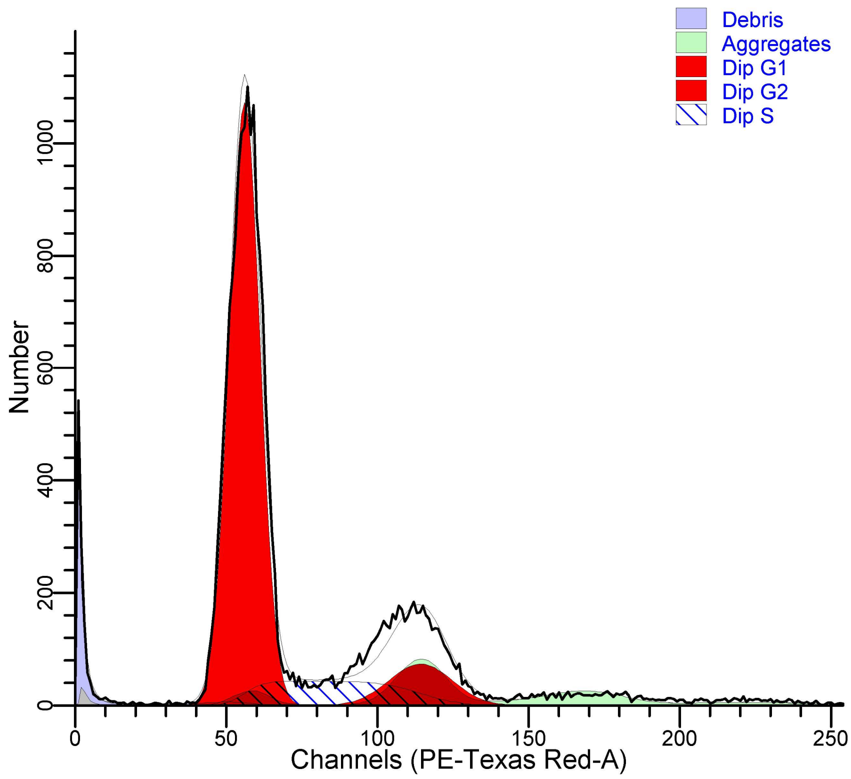

File analyzed: s7_GR2_005.fcs
Date analyzed: 12-Jul-2020
Model: 1DA0n_DSD
Analysis type: Manual analysis

Ploidy Mode: First cycle is diploid

Diploid: 100.00 %
Dip G1: 75.62 % at 56.01
Dip G2: 10.62 % at 114.26
Dip S: 13.76 %   G2/G1: 2.04
%CV: 9.08

Total S-Phase: 13.76 %
Total B.A.D.: 9.96 %

Debris: 5.72 %
Aggregates: 15.01 %
Modeled events: 22838
All cycle events: 18103
Cycle events per channel: 306
RCS: 2.996

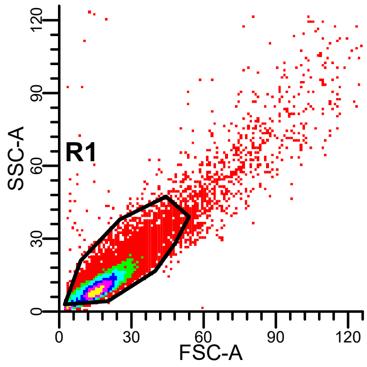

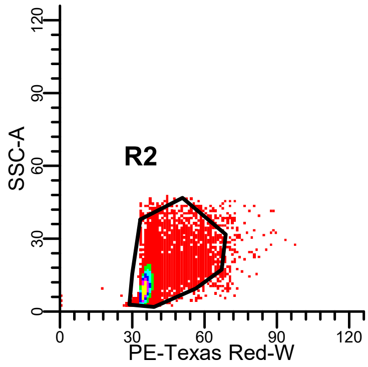

Supplement: Supplementary file 1 [file cells-10-00423-s001.zip › cells-1048437-Supplementary Materials/S2/siR-MSTN and siR-NC/siR-MSTN-2.pdf]
