# Supplementary material for: MicroRNA-27b-3p Targets the Myostatin Gene to Regulate Myoblast Proliferation and Is Involved in Myoblast Differentiation
Source: Cells. 2021 Feb 17;10(2):423. doi: 10.3390/cells10020423 (PMC7922189; doi:10.3390/cells10020423)
Supplement: Supplementary file 1 [file cells-10-00423-s001.zip › cells-1048437-Supplementary Materials/S2/siR-MSTN and siR-NC/siR-MSTN-3.pdf]

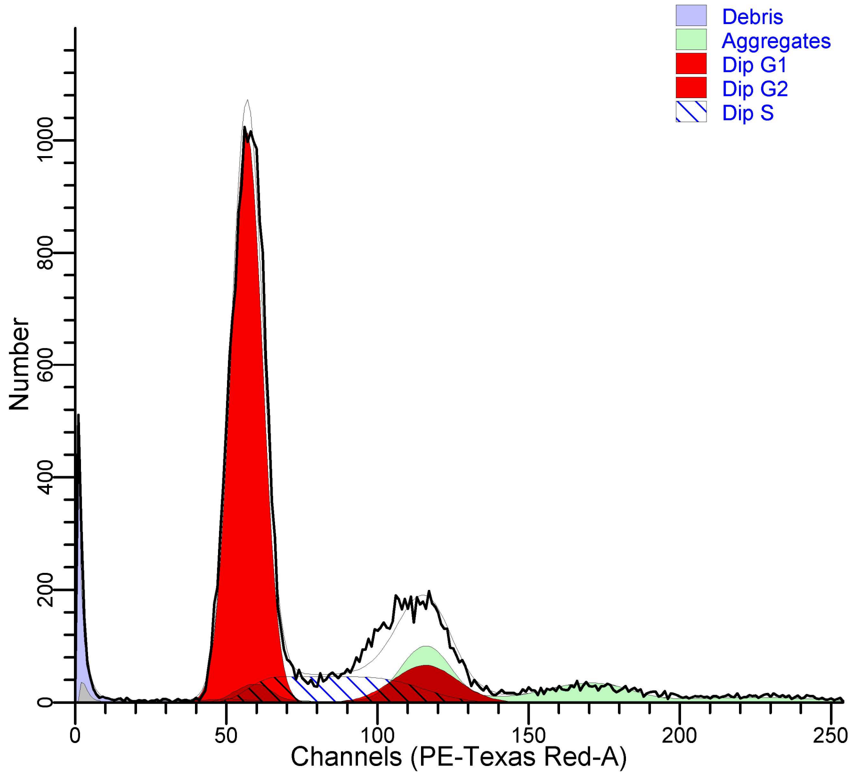

File analyzed: s7_GR3_006.fcs
Date analyzed: 12-Jul-2020
Model: 1DA0n_DSD
Analysis type: Manual analysis

Ploidy Mode: First cycle is diploid

Diploid: 100.00 %
Dip G1: 74.66 % at 56.71
Dip G2: 9.92 % at 115.69
Dip S: 15.42 %   G2/G1: 2.04
%CV: 9.18

Total S-Phase: 15.42 %
Total B.A.D.: 11.35 %

Debris: 5.38 %
Aggregates: 19.19 %
Modeled events: 23546
All cycle events: 17761
Cycle events per channel: 296
RCS: 2.878

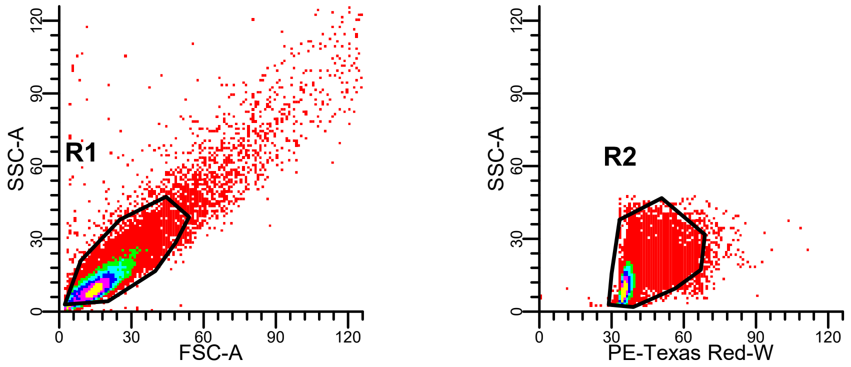

ModFitLT V3.1(Win32)
