# Supplementary material for: MicroRNA-27b-3p Targets the Myostatin Gene to Regulate Myoblast Proliferation and Is Involved in Myoblast Differentiation
Source: Cells. 2021 Feb 17;10(2):423. doi: 10.3390/cells10020423 (PMC7922189; doi:10.3390/cells10020423)

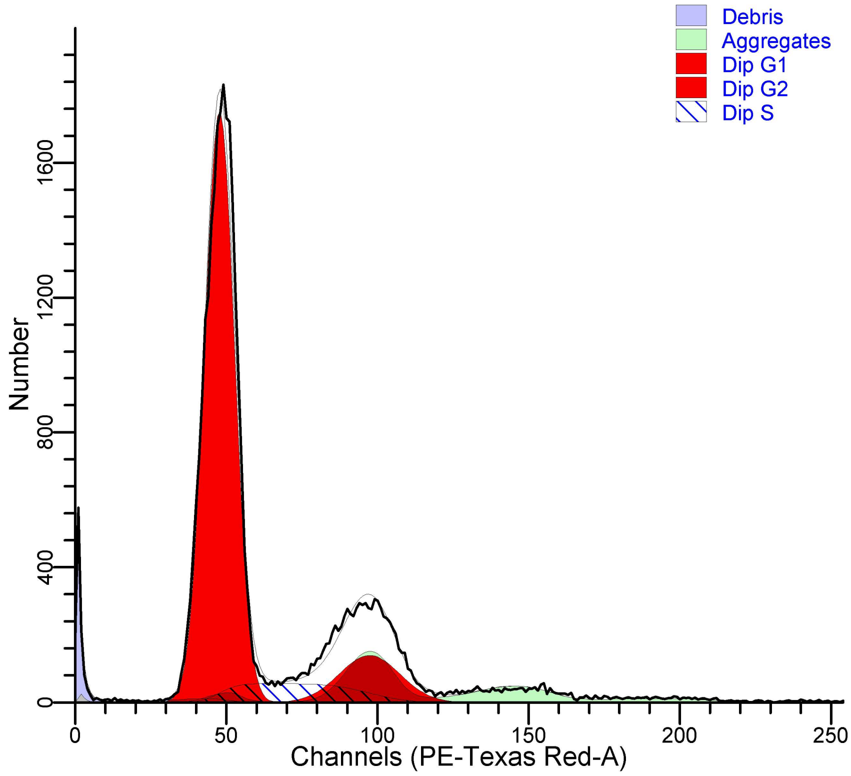

File analyzed: s7_NC1_001.fcs
Date analyzed: 12-Jul-2020
Model: 1DA0n_DSD
Analysis type: Manual analysis

Ploidy Mode: First cycle is diploid

Diploid: 100.00 %
Dip G1: 77.53 % at 47.71
Dip G2: 12.54 % at 97.33
Dip S: 9.93 %   G2/G1: 2.04
%CV: 10.30

Total S-Phase: 9.93 %
Total B.A.D.: 9.94 %

Debris: 3.89 %
Aggregates: 16.11 %
Modeled events: 34952
All cycle events: 27960
Cycle events per channel: 552
RCS: 3.409

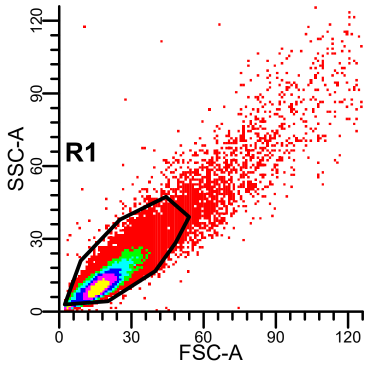

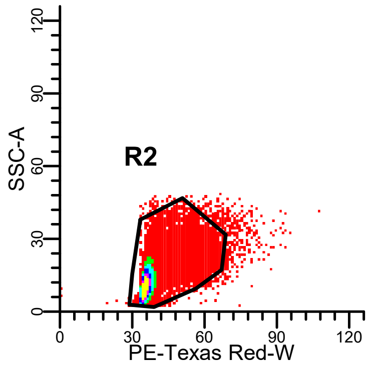

Supplement: Supplementary file 1 [file cells-10-00423-s001.zip › cells-1048437-Supplementary Materials/S2/siR-MSTN and siR-NC/siR-NC-1.pdf]
