# Supplementary material for: MicroRNA-27b-3p Targets the Myostatin Gene to Regulate Myoblast Proliferation and Is Involved in Myoblast Differentiation
Source: Cells. 2021 Feb 17;10(2):423. doi: 10.3390/cells10020423 (PMC7922189; doi:10.3390/cells10020423)

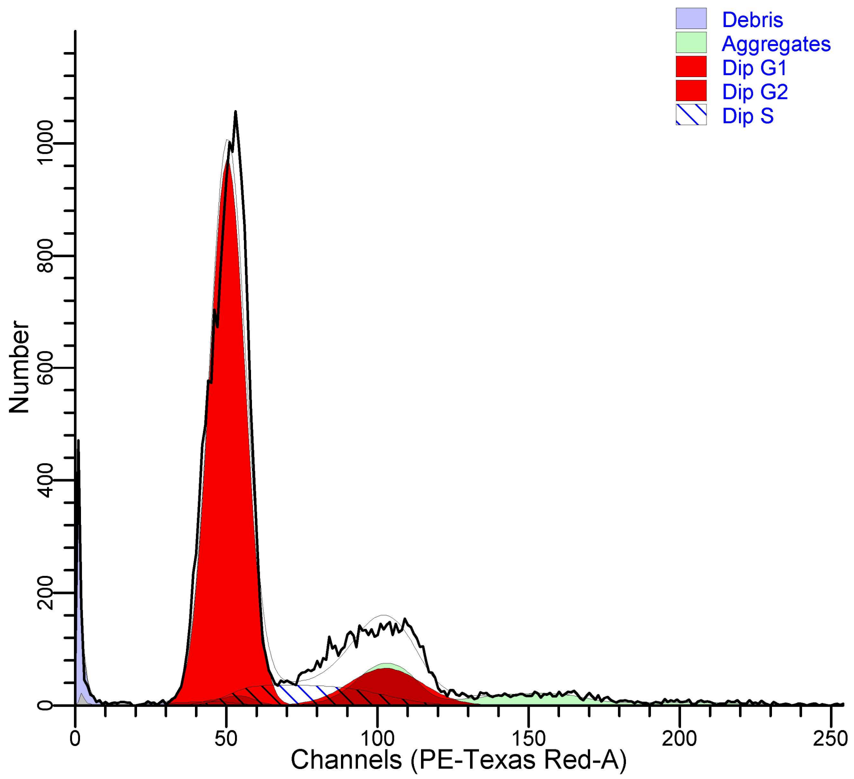

File analyzed: s7_NC2_002.fcs
Date analyzed: 12-Jul-2020
Model: 1DA0n_DSD
Analysis type: Manual analysis

Ploidy Mode: First cycle is diploid

Diploid: 100.00 %
Dip G1: 78.70 % at 50.30
Dip G2: 10.92 % at 102.62
Dip S: 10.38 % G2/G1: 2.04
%CV: 11.68

Total S-Phase: 10.38 %
Total B.A.D.: 9.35 %

Debris: 4.66 %
Aggregates: 14.62 %
Modeled events: 22543
All cycle events: 18195
Cycle events per channel: 341
RCS: 4.164

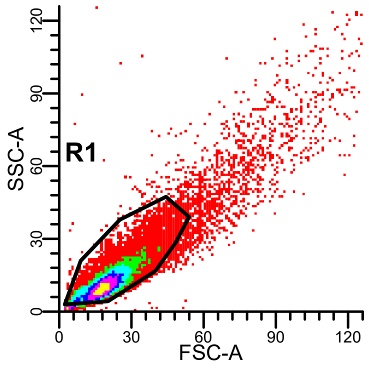

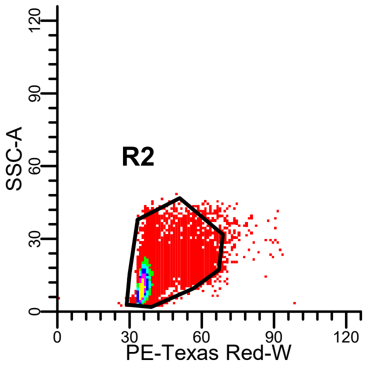

Supplement: Supplementary file 1 [file cells-10-00423-s001.zip › cells-1048437-Supplementary Materials/S2/siR-MSTN and siR-NC/siR-NC-2.pdf]
